# Developing evaluation index system for “new medicine” construction in Chinese medical schools—meta-ethnography and Delphi-AHP approach

**DOI:** 10.3389/fmed.2025.1717165

**Published:** 2026-01-07

**Authors:** Dandan Zheng, Norlizah Che Hassan, Norliza Ghazali, Yuee Chai, Dan Zhang, Wanru Lyu

**Affiliations:** 1 Department of Science and Technology, Guizhou Medical University, Guiyang, China; 2 Department of Foundation of Education, Faculty of Educational Studies, University Putra Malaysia, Serdang, Malaysia; 3 School of Medicine and Health Management, Guizhou Medical University, Guiyang, China

**Keywords:** Delphi-AHP, evaluation index system, medical schools, meta-ethnography, new medicine

## Abstract

**Objective:**

This study intends to construct a scientific, comprehensive and operable evaluation index system for the constructing achievements of “New Medicine” in medical schools and contribute to promoting the innovative development of medical education in China.

**Methods:**

The study adopted a sequential mixed-methods design. An initial indicator system was first constructed through meta-ethnographic analysis, which was subsequently refined through two rounds of Delphi consultations by 12 experts. In the final stage, the relative weights of the indicators were quantified and validated using the Analytic Hierarchy Process (AHP).

**Results:**

The final evaluation index system includes 11 first-level indicators and 37 second-level indicators. Among them, the top three first-level indicators with the highest weight coefficients are the reform of the curriculum system (0.1790), the education and training of medical students (0.1550), and the optimization and adjustment of the structure of disciplines and majors (0.1530), emphasizing the fundamental role of these three in school’s medical education, while the stakeholder feedback mechanism (0.0190) is used as a supplementary element.

**Conclusion:**

This study constructs an scientifically grounded evaluation index system for the development of “New Medicine” in medical schools, addressing the current lack in existing research of quantifiable measures for assessing medical innovation education in the new era. Meanwhile, it can serve as a reference for relevant researches on medical education reform, and provide evaluation benchmark for education regulatory departments, policy makers, as well as medical school administrators.

## 1 Introduction

### 1.1 Background

Medical schools not only bear the core mission of cultivating high-quality healthcare and public health professionals (1, 2) but also serve as crucial platforms for medical science innovation, playing an irreplaceable role in tackling major challenges in disease prevention, control and health-related scientific issues (3, 4). At present, pressures on healthcare and public health are intensifying due to severe global aging population, disease spectrum changes as well as chronic disease burden (5). Under such circumstances, medical schools are entrusted with even greater responsibilities and more formidable missions in advancing medical education, research, and healthcare delivery (6, 7). Moreover, the introduction of bio-psycho-social medical model has led to a more systemic understanding of health and life has emerged, accompanied by a cognitive shift in focus from treatment to prevention (8, 9). Meanwhile, it provides new insights for health and disease causes from multiple perspectives and further explores multifaceted approaches to disease treatment and prevention, characterized by holism, systematism, and plurality (9, 10).

In addition, within the broader context of the Fourth Industrial Revolution, technological advancements are transforming medical research and practice at an unparalleled speed and scale, further accelerating trend of interdisciplinary integration (11). The convergence of medicine with engineering, natural science, information science, as well as humanities and social sciences has not only expanded the theoretical frontiers of medical research, but also provided a new methodological perspective and innovative path for addressing complex health problems (12). Against this backdrop, as the supplier of healthcare workforce, medical educational system is confronted with unprecedented challenges: it must not only respond to the increasingly diversified and high-quality demands of the healthcare market, but also be committed to cultivating high-level medical talents equipped with interdisciplinary knowledge, excellent clinical professional skills, and a deep sense of humanistic care (13).

To date, global medical education has undergone the stage of establishing a standardized modern medical education system represented by the Flexner Report in the United States (14). This was followed by a shift toward integrated education, characterized by pedagogical reform through problem-based learning (PBL) and the emphasis on cultivating clinical competence and later the phase of establishing a synergistic development of medical education system and health service system promoting the accessibility and equal development of high-quality medical services worldwide (15). These three major revolutions have laid the basic foundation for medical education system (16). Of course, the current global medical education reflects a state of coexistence of three generations of medical education. Most countries still retain the traditional discipline-based lecture methods, while only a few countries have advanced to the third generation of reform.

In China, the reform of medical education since the beginning of the 21st century has mirrored the three waves of reform in international medical education (17). During the first decade of the 21st century, two representative studies had a profound impact on China’s medical education policy: *Research on the Reform of China’ Medical Education Management System and Academic Degree System* (2003) and *Research and Practice on the Construction of a Scientific System of Medical Education in China* (2007). These works addressed critical issues including management mechanisms, degree structures, quality assurance, lifelong education and talent cultivation models. Their focus corresponded to the international shift from the first generation of medical education—characterized by discipline-based and lecture-centered teaching—to the second generation, which emphasized problem-based learning (PBL) and the cultivation of clinical competence.

During the second decade, medical education reform in China has deepened substantially, especially in clinical medical education, where the nationwide adoption of the “5 + 3” model marked a fundamental shift in both the structure and the mode of physician training (18, 19). During this period, two initiatives had a major impact on the reform of medical education in China: the international landmark report on the third generation of medical education reform published in 2010, which featured contributions from Chinese scholar Ke Yang (15) and the subsequent “Innovative Project on Medical Education Reform in China for the 21st Century” that she led in 2011 (17). This report systematically analyzed the opportunities and challenges facing medical education in the new era and proposed 16 policy recommendations, which generated widespread attention and debate within the field (20).

However, at present, artificial intelligence technology is not only changing how knowledge is produced and transmitted, but also profoundly reshaping cognitive approaches (21). The traditional disciplines with single knowledge field and clear boundaries will be replaced by newly integrated fields, which will drive a comprehensive reform and reshape the whole process of medical education and teaching, the era of the fourth medical education reform has been unfolding (22).

In light of this context, the Central Committee of the Communist Party of China and the State Council issued a key document on education reform and development in the new era in 2018, formally introducing the concept of “New Medicine” for the first time, with subsequent policies being launched accordingly. Chinese government has made the construction of “New Medicine” an important task in medical education reform in response to the evolving healthcare service models and shifting public perceptions of life and health driven by the new wave of scientific and technological revolution (23).

As the primary entities implementing and benefiting from “New Medicine” initiatives, medical schools are centered on breaking down the barriers of traditional medical education. They emphasize interdisciplinary integration and practice-oriented approaches, striving to cultivate high-quality medical talents who are adaptable to future medical development trends and possess both humanistic qualities and professional capabilities (24, 25). Despite increasing discussion about the medical education innovation, for medical schools, how to establish medical talents cultivating and developing system remains an open question.

### 1.2 Research gap, scope and aim

Regarding the current exploration of “New Medicine” construction, related studies have not only focused on the connotation analysis of “New Medicine,” strategic significance, construction paths, organizational systems and so forth (23, 26, 27), but also attempted to explore the adjustment of academic and professional structures, reform of talent cultivation models (27), construction of curriculum systems (28), and teaching approach reform, all within the context of the “New Medicine” background (29).

Besides, practical explorations have been conducted in interdisciplinary areas including medicine-engineering, medicine-literature, medicine-information, etc. The application of artificial intelligence (AI), big data, and other technologies have played increasingly important role in disease diagnosis, treatment, and prevention (30). Specifically, AI is transforming medical education with innovative tools like virtual reality (VR), augmented reality (AR), adaptive learning platforms, and AI-powered assessments, which are gaining recognition for their potential to improve diagnostic accuracy, clinical decision-making, and personalized learning experiences (31). Furthermore, the continuous development of narrative medicine based on empathy theory, primarily based on the interdisciplinary integration of “New Medicine” and “New Liberal Arts,” has demonstrated promising effects in enhancing doctor-patient communication and promoting disease diagnosis and rehabilitation (32).

In summary, while existing researches has mainly focused on policies interpretation and the specific reform experiences in certain professional domains, systematic evaluation of the effectiveness of “New Medicine” construction in medical schools has received insufficient attention.

From the perspective of higher-education quality evaluation, China started the development of systematic assessment mechanisms relatively later than Western countries and existing studies about medical education evaluation system remains underdeveloped and fragmented. Some studies focus primarily on internal quality assurance within medical schools. For instance, Zhang et al. (2025) developed an evaluation index system for clinical teaching quality for international medical students in China (33). Ji and Cui (34) synthesized global practices in medical humanities education and proposed an evaluation system for medical schools’ humanities curricula.

In addition, the Science and Technology Evaluation Metrics (STEM) and the Accumulative Science and Technology Evaluation Metrics (ASTEM) issued by the Chinese Academy of Sciences mainly target scientific research performance and technological innovation capacity. These frameworks are suitable for assessing the research strength, disciplinary development, and long-term growth potential of medical universities or teaching hospitals, rather than the broader educational dimensions. Other studies have focused on developing evaluation systems for the holistic quality of medical students, emphasizing competencies and educational outcomes (35).

Moreover, although some researches have proposed comprehensive evaluation frameworks for medical education, they mainly focused on traditional medical education, typically covering generic dimensions such as basic teaching quality, institutional qualifications, faculty capacity, student training quality, graduate management, and research capability (36–38). However, these frameworks do not incorporate dimensions central to the construction of “New Medicine,” such as innovation capacity, interdisciplinary integration, and the coordinated development of clinical practice, scientific research, and teaching. These gaps highlight the necessity of establishing a more targeted evaluation index system aligned with the goals and characteristics of “New Medicine” construction.

Hence, due to the lack of a unified, comprehensive, systematic, multidimensional, and quantifiable evaluation index system which can assess the progress of “New Medicine” initiatives, the quality of talent cultivation, and the structural transformation of the medical education system, this study aims to fill the significant gap in current “New medicine” research regarding “how to evaluate,” thereby promoting the high-quality development of medical education.

## 2 Methods

### 2.1 Research design

This research adopted sequential mixed-methods design by employing relevant policy documents and current domestic and international research findings as the basis for analysis. Specifically, the meta-ethnography, Delphi and Analytic Hierarchy Process (AHP) methods are employed in sequence to construct an evaluation index system for medical schools’ “New Medicine” construction in China.

### 2.2 Data collection and analysis procedures

This study first employs meta-ethnography method to conduct an experimental pre-selection of evaluation indicators for “New medicine” construction in medical schools. Meta-ethnography is a mature method which does not merely summarize existing studies but generates new insights by extracting the core concepts or factors of related researches and then through comparing and analyzing their similarities and differences to form a novel synthesis (39). Hence, the preliminary construction of the evaluation index system is carried out by using the methods and procedures of theme extraction and coding specified by meta-ethnography.

**Figure 1:**
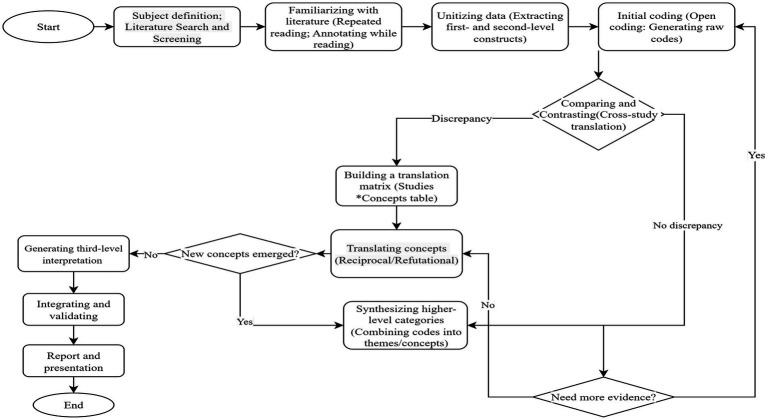
Flowchart of preliminary construction of evaluation index system.

At first, the research questions and themes were clarified, the retrieval strategy, inclusion/exclusion criteria were formulated, and the process was recorded according to PRISMA (preferred reporting items for systematic reviews and meta-analysis) and CASP (critical appraisal skills programme) principles to identify and screen the policy documents, papers, books and other literature on the “New Medicine” construction. Then, the final original documents were read and analyzed in detail and repeatedly, and preliminary ideas and potential comparison points were recorded. Next, after the stages of dividing units, initial coding, comparison, translation, summarizing high-level categories and establishing translation matrix, reciprocal and refutational translation, the evaluation dimension and supporting first-level indicators as well as specific second-level indicators of the “New Medicine” construction effect evaluation index system of China’ medical schools are finally formed (40, 41). The specific process and procedures are illustrated in Figure 1.

Next, the Delphi method was used to further discuss and determine the preliminary evaluation index system. It is a very important method among the expert survey methods. With the knowledge and experience of experts, the characteristics and development laws of the research subject were found based on experts’ comprehensive analysis, and after simple statistics and inference, the final prediction is carried out (36). In detail, firstly, the preliminary evaluation indicators were designed into a survey questionnaire, and background materials relevant to the survey topic were gathered, as well as preparatory work were completed prior to the survey. The second step is expert panel selection. Because this study focused on “New medicine” construction, the expert panel composition adhere to the principle of heterogeneity of multidisciplinary background and multiple experience, balancing the composition of theoretical researchers and practical experts, and ensuring that the selected experts are representative and authoritative in the field of medical education and can complete 2–3 rounds of consultation (42). Thirdly, the preliminary index system was repeatedly consulted by sending questionnaires to experts, and the final expert opinions were statistically processed and analyzed, including expert authority coefficient, expert opinion concentration degree, consistency test of expert evaluation results (Kendall’s coordination coefficient and index variation coefficient). According to the statistical results, the level and content of the evaluation index of the “New medicine” construction effect in medical schools were established.

Finally, AHP is used to get the weight of the evaluation index of the “New medicine” construction effect in medical schools. AHP is a method which involves both qualitative and quantitative, systematic and hierarchical analysis proposed by Thomas L. Saaty in the 1970s. Because of its practicability and effectiveness in dealing with complex decision-making problems, it has been paid attention to all over the world (43).

The specific steps are elaborated as follows: firstly, a hierarchical structure model was constructed and indicators and hierarchical structure were established. Then the elements of each layer are compared in pairs, and the judgment matrix is constructed by using the 1–9 scaling method. Specifically, assuming that the evaluation objective was A, the j elements subjected by A were W1, W2, …, Wn, For i, j = 1, 2, …, *n*. a_ij_ was used to represent the ratio of influence of *W*_i_ and *W*_j_ on A. The value of a_ij_ is the comparison of the importance between *W*_i_ and *W*_j_ according to the expert evaluation, and then make pairwise comparison to construct the comparison matrix, that is, the judgment matrix.


$$A=(\begin{array}{cccc} \frac{w1}{w1} & \frac{w1}{w2} & \ldots & \frac{w1}{\mathrm{wn}}\\ \frac{w2}{w1} & \frac{w2}{w2} & \ldots & \frac{w2}{\mathrm{wn}}\\ \vdots & \vdots & \vdots & \vdots \\ \frac{\mathrm{wn}}{w1} & \frac{\mathrm{wn}}{w2} & \ldots & \frac{\mathrm{wn}}{\mathrm{wn}} \end{array})$$

If *W*_i_ is more important than *W*_j_, the *W*_ij_ value would be bigger. At the mean time, *W*_ij_ = 1/*W*_ij_, indicating that j is less important than i. Then Satty’s 9-scale method was used to find the corresponding ratio value.

Secondly, the maximum eigenvalue λ_max_ of the judgment matrix P and the corresponding eigenvector $m={\left(m_{1,}m_{2,}\ldots m_{n}\right)}^{T}$ were solved. The consistency of judgment matrix $P={\left(a_{\mathrm{ij}}\right)}_{n\times n}\;$ was needed to be tested by using the consistency indicator $\mathrm{CI}=\frac{\lambda \max‐n}{n‐1}$ and consistency ratio, $\mathrm{CR}=\frac{\mathrm{CI}}{\mathrm{RI}}$ , (RI is the average random consistency indicator). When CI < 0.1, it implied that the constructed judgment matrix P had the satisfactory consistency. Then, for the judgment matrix that conforms to the consistency test and does not need to be modified, the relative weight of the comparison elements is calculated, and the characteristic item quantity is normalized, that is, according to the formula.

$\mathrm{wi}=\frac{\mathrm{Mi}}{\sum_{j-1}^{n}\mathrm{Mj}}$ , transforming the eigenvector corresponding to the maximum eigenvalue to obtain a new normalization vector $w={\left(w_{1,}w_{2,}\ldots w_{n}\right)}^{T}$ . Finally, the individual weight and composite weight of each indicator were obtained.

## 3 Results

### 3.1 Preliminary framework of “new medicine” construction evaluation indicator system

Firstly, based on the research topic, the researchers conducted searches and collected data from CNKI, WOS, and relevant policy documents from 1st, January, 2000 to 31st, December, 2024. After preliminary screening, this yielded a total of 361 retrieved articles and 7 collected policy documents.

**Figure 2:**
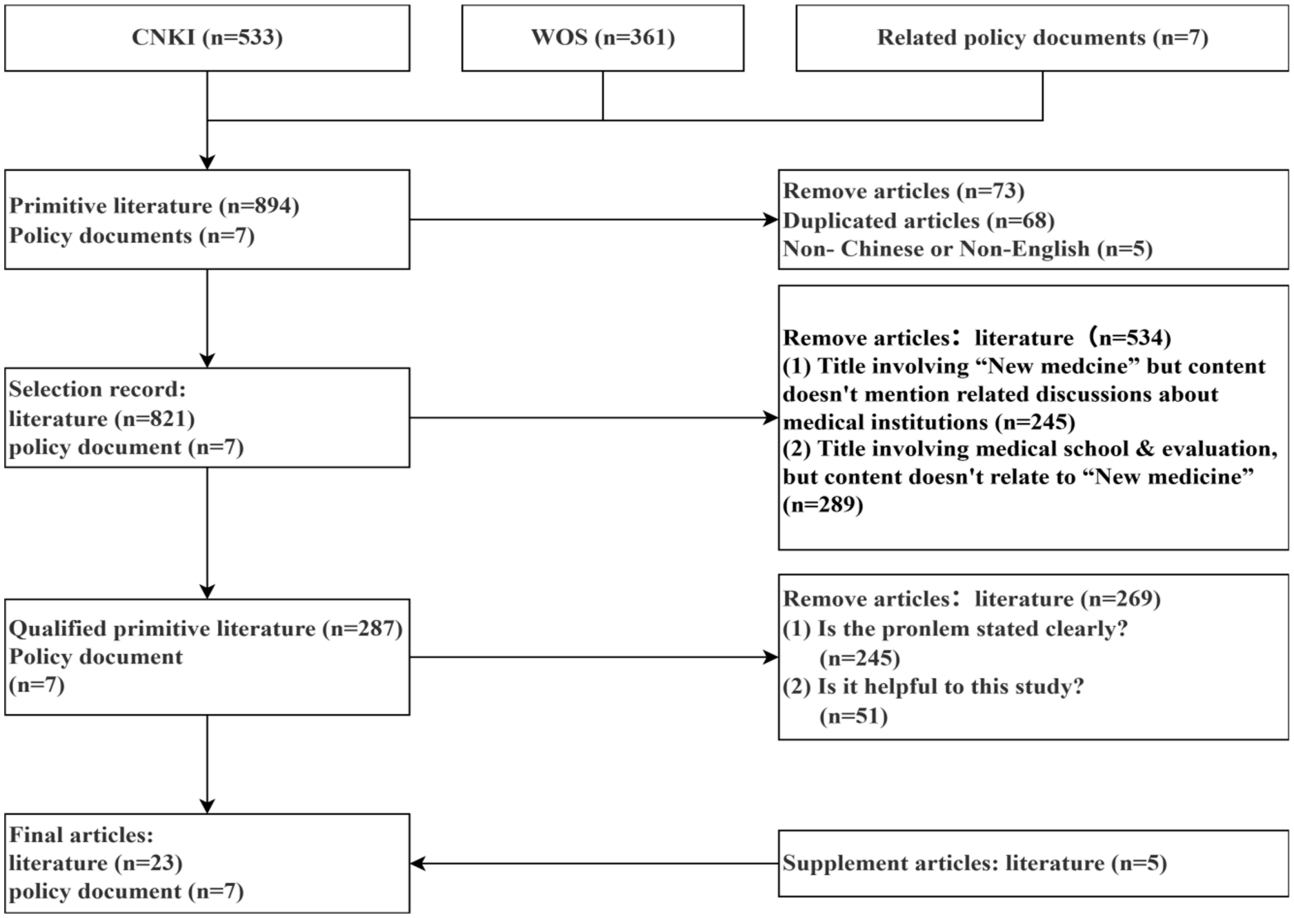
Result of literature evaluation and screening process.

According to the PRISMA and CASP principles, the research team conducted a task-divided, cross-evaluation screening process, which resulted in the retention of 7 policy documents and 23 research articles for further in-depth review. Among them, five articles were later incorporated as supplements, which were singled out by other researchers during the cross-evaluation of the previously excluded literature. Upon collective discussion, these were included as supplementary materials, resulting in a final corpus of 30 documents. The selection process is detailed in Figure 2.

Next, through detailed reading, analysis, and comparison of the identified 30 original documents, key themes were extracted. The core themes were then mutually translated and synthesized, resulting in an overall explanatory framework for the indicator system, which includes 10 primary indicators and 34 secondary indicators.

### 3.2 Basic information of the expert

A total of 12 experts were selected for this study and to ensure the comprehensiveness of the consultation opinions, the members include front-line lecturers and researchers from various disciplines in medical schools, clinical experts from affiliated hospitals, as well as medical education administrators. All of them held a doctoral degrees or a senior professional title in relevant fields, and possess rich experience and a strong research background in the development of medical schools and the students training, and the composition of experts panel and some key information of them can be seen in Table 1.

**Table 1:** Basic information on experts.

Basic information		No. of people (*N* = 12)	Percentage
Gender	Male	5	41.7%
	Female	7	58.3%
Professional role	Clinical expert	2	16.7%
	Medical researcher	3	25.0%
	Medical education expert	3	25.0%
	Medical school faculty	2	16.7%
	Medical school administrators	2	16.7%
Years engaged in medical education	5–10 years	5	41.7%
	10–15 years	4	33.3%
	15–20 years	2	16.7%
	More than 20 years	1	8.3%
Highest education level	Master	2	16.70%
	PHD	10	83.30%
Professional titles	Associate professor	5	41.70%
	Full professor	7	58.30%

The participants were predominantly PHD holders (83.30%; *n* = 10), with only two experts having master degree (16.70%, *n* = 2). The experts’ areas of expertise are relatively well-balanced, clinical medicine experts (16.7%, *n* = 2), medical researchers (25.0%, *n* = 3), medical education experts (25.0%, *n* = 3), medical teachers (16.7%, *n* = 2). All experts had more than 5 years’ working experience and held senior professional titles, moreover, most of them were professors (58.30%, *n* = 7).

### 3.3 Expert consultation result

Expert consultations were conducted by distributing online questionnaires to experts, which consisted of three sections. The first section employed a 5-point Likert scale (where 5 = very important, 4 = relatively important, 3 = moderately important, 2 = unimportant, and 1 = completely unimportant) to assess the importance, feasibility, and relevance of each indicator. The second section included an open-ended comments and revisions section, project addition section and remarks section, to facilitate experts in providing their comments and suggestions. The third section was a self-assessment form to evaluate the experts’ authority on the subject.

Two rounds of expert consultations were conducted in this phase. Round 1 consultation results showed that the mean score for the importance of the indicators is 4.038 with all values exceeding 3.5, The coefficient of variation (CV) for each indicator was below 0.25, indicating relatively low score dispersion among experts. However, since the value of Kendall’s coefficient of concordance (*W*) is supposed to be between 0 and 1, and the higher the value means that the opinion of the experts is more consistent and the coordination of the items is better, while the first round consultation result showed that the Kendall’s W was only 0.194, suggesting a relatively low level of consensus among the experts. Based on experts’ feedback, three additional indicators were added, and the descriptions of certain indicators have been modified to enhance their operability and applicability.

Thus, the second round of expert inquiry questionnaires includes a total of 11 primary indicators and 37 secondary indicators. It was redistributed to the same 12 experts, and all 12 valid questionnaires were returned within 1 week, yielding a 100% response rate. The analysis results showed that the CV for all indicators remained below 0.25, and the Kendall’s W was 0.433, which is acceptable as it fell between 0.4 and 0.5 and demonstrate that expert opinions were generally consistent. Besides, the difference was statistically significant by chi-square text (*p* < 0.001). The detailed information was presented in Tables 2 and 3 respectively.

**Table 2:** The result of expert opinions’ concentration degree.

Consultation round	Hierarchical level	Importance value	Full-score rate (%)
Round 1	First-level	3.58–4.42	33.3–50.0%
	Second-level	3.58–4.83	33.3–58.3%
Round 2	First-level	4.26–4.65	33.3–58.3%
	Second-level	4.13–4.89	33.3–58.3%

**Table 3:** The result of expert opinions’ coordination degree.

Consultation round	Hierarchical level	Coefficient of variation (CV)	Kendall’s W	*χ* ^2^	*p*
Round 1	First-level	0.15–0.22	0.194	99.957	<0.001
	Second-level	0.08–0.22			
Round 2	First-level	0.11–0.21	0.433	239.157	<0.001
	Second-level	0.07–0.20			

Additionally, this study employed an expert self-assessment approach to assess expert authority coefficient (Cr) and the distribution of the values ranges between 0.7 and 1.0, with an average of 0.77. The authoritative coefficients of each expert are shown in Table 4.

**Table 4:** Experts’ authoritative coefficient.

Expert code	Familiarity coefficient (Ca)	Criteria for judgment				Basis for judgment (Cn)	Authority coefficient (C_r_)
		Theoretical analysis	Practical experience	Referencing domestic and international sources	Intuitive choice		
1	0.75	0.3	0.5	0.1	0.1	1	0.875
2	0.5	0.3	0.4	0.1	0.1	0.9	0.7
3	0.5	0.2	0.4	0.1	0.1	0.8	0.65
4	0.75	0.3	0.5	0.1	0.1	1	0.875
5	0.75	0.2	0.5	0.1	0.1	0.9	0.825
6	0.5	0.2	0.5	0.1	0.1	0.9	0.7
7	0.75	0.3	0.4	0.1	0.1	0.9	0.825
8	0.75	0.2	0.5	0.1	0.1	0.9	0.825
9	0.5	0.2	0.5	0.1	0.1	0.9	0.7
10	0.5	0.3	0.5	0.1	0.1	1	0.75
11	0.25	0.3	0.4	0.1	0.1	0.9	0.575
12	1	0.1	0.5	0.1	0.1	0.8	0.9
Mean	0.625	0.24	0.47	0.1	0.1	0.91	0.77

### 3.4 Weights distribution of “new medicine” constructing effect evaluation index

Based on the 11 primary and 37 secondary indicators derived from the first two rounds of expert consultation, a hierarchical analytical model was constructed, followed by the construction of a pairwise comparison judgment matrix. Next, after hierarchical single sorting, namely, ranking after determining the indicator weights, consistency verification was conducted, the formula is CR = CI/RI, The calculating results showed that CR < 0.1, indicating that each judgment matrix satisfied the consistency criterion (see Table 5). Meantime, the weight coefficient of each index was obtained and the weight coefficients of all indicators were presented in Supplementary Table S1.

**Table 5:** The result of consistency test of each judgment matrix.

First-level indicator				Second-level indicator			
Indicator	λmax	CI	CR	Indicator	λmax	CI	CR
A-K	11.6123	0.0485	0.03190	A1-A3	3.0293	0.01465	0.0282
				B1-B4	4.1176	0.03919	0.044
				C1-C3	3.0016	0.00079	0.0015
				D1-D5	5.0245	0.00613	0.0055
				E1-E5	5.1541	0.03853	0.0344
				F1-F4	4.0032	0.00106	0.0012
				G1-G2	2	0	0
				H1-H4	4.0111	0.00368	0.0041
				I1-I3	3.0003	0.00016	0.0003
				J1-J3	3.0044	0.0022	0.0042
				K1-K1	1	0	0

## 4 Discussion

### 4.1 The characteristics of evaluation index system for “new medicine” construction in Chinese medical schools

The evaluation index system constructed in this study embodies the features of comprehensiveness, scientific validity, diversification and reliability. It aligns with the core mission proposed in “New Medicine” initiative by the Chinese Ministry of Education in 2018, to build a modern medical education system for the future through technological innovation, interdisciplinary integration and the transformation of talent training models (44).

The “New” in “New Medicine” reflects a dual focus. It involves leveraging cutting-edge technology to innovate the medical education paradigm and critically re-examining the current state of medical education to initiate a new chapter of reform. The goal is not only cultivating high-level innovative medical compound talents to solve the “bottleneck” problem, but also cultivating high-quality primary healthcare physicians who can save lives and heal the wounded, have high medical ethics, as well as meet the basic health needs of 1.4 billion people in China (45, 46). The evaluation index system for the construction of “New Medicine” in this study is to evaluate the state of the development of medical schools in all aspects under the relevant construction elements and requirements of “New Medicine,” which has strong applicability and practicality.

Moreover, this study collected relevant literature, data, government policy documents and reports from home and abroad, and strictly followed the seven steps of meta-ethnography to construct the draft of the index system (47). This process involved five experienced researchers, which minimized the omission of relevant indicators and enhanced the validity and credibility of the draft. Furthermore, the preliminary system underwent two rounds of Delphi consultation with 12 experts from various domains of medical education. Furthermore, the preliminary system underwent two rounds of Delphi consultation with 12 experts from various domains of medical education, which further proved the systematization, authority and scientificity of the index system.

Finally, the subjects of the index system is diversified. It can be used by the educational regulatory authorities to evaluate the operation of the school in all aspects of the construction of “New Medicine” among various types of medical schools, and can also be used by school administrators, teachers and researchers, as well as students, conducting multi-subject evaluation and mutual evaluation between schools to help improve the school’s own construction and development and the quality of talent training.

### 4.2 Analysis about the weights distribution of “new medicine” construction evaluation index system

AHP is an effective decision-making approach that quantifies expert subjective judgments to produce scientific results. After pair-wise comparison and the establishment of the weights of indicators at all levels, it is found that curriculum system reform plays a leading role in medical schools’ “New medicine” construction evaluation. The first-level indicators ranking in the top five by weight coefficients are, respectively, Curriculum System Reform (0.1790), Medical Student Education and Training (0.1550), Optimization and Adjustment of the Structure of Academic Programs (0.1530), Faculty Competence Development (0.1340), Discipline System Construction (0.1020).

Firstly, the curriculum system is an organic whole, including curriculum types, curriculum content, curriculum forms, curriculum implementation and evaluation, etc. As the primary vehicle for implementing “New Medicine” concepts and plans, it serves as the foundation for other dimensions, which is why most experts regarded it as the most critical component.

Secondly, since the focus and ultimate goal of the construction of “New Medicine” in China is to cultivate high-level, high-quality, internationally outstanding medical talents and future medical leaders with both medical ethics and professional competence, this indicator also gained higher weight coefficients among others.

Thirdly, the establishment and development of programs should be closely aligned with market demand. Given the shift from the biomedical model to the bio-psycho-social model, coupled with the ongoing scientific and technological revolution, the content and mode of medical education must be transformed and adjusted in a timely manner.

Fourthly, teachers are the key role in medical education, and they shoulder the dual tasks of cultivating students and scientific research innovation, so the faculty competence development also constitute a relatively large proportion among all indicators.

Finally, the innovation of the knowledge system in the construction of “New Medicine” must rely on a robust disciplinary system. The “New Medicine” initiative calls for breaking through the disciplinary boundaries of the traditional knowledge system to meet the major strategic needs of national development and the cutting-edge scientific and technological challenges, as well as the actual needs of public health. It emphasizes deep integration with other fields—such as sciences, agriculture, humanities, and engineering—and encourages the sustainable development of emerging interdisciplinary areas like precision medicine, which serves as a fundamental guarantee for progress.

### 4.3 The significance of constructing “new medicine” construction evaluation index system for medical schools

The construction of an evaluation index system for medical institutions is a complex and multi-dimensional process, aiming to objectively and scientifically assess the educational quality and development potential of medical schools, thereby providing robust basis to guide their development (48). However, the existed studies mainly emphasize on the traditional and conventional medical education evaluation, such as construction of evaluation indicators for specific courses in different disciplines (49, 50), faculty competence evaluation (51), the clinical and research competence evaluation index construction of medical students and so on (52). Little attention has been paid to constructing a systematic evaluation index system for “New Medicine” that incorporates the characteristics of medical education in the context of the new era.

To address this gap, this study drew on the characteristics and requirements of the paradigm transformation of medical education against the backdrop of the new technological revolution to construct a comprehensive and well-targeted evaluation index system for medical institutions (53). By aligning indicators with the core principles of “New Medicine,” medical schools can form a clear reform orientation in the aspects of interdisciplinary integration, pedagogical innovation, and the cultivation of clinical competence and research capacity, so as to promote the cultivation of compound and innovative medical talents. This is of great significance for shifting away from traditional disciplines-based and lecture-based models toward training innovative, interdisciplinary, and practice-oriented medical talents who can meet the evolving demands of healthcare systems.

Methodologically, although the construction of the existing evaluation index system mostly adopts Delphi and AHP, there are few studies using the meta-ethnography method to comprehensively integrate and form the initial framework of the evaluation index, which made the establishment of the evaluation index system of this study more systematic and scientific (54).

## 5 Conclusion

At first, the “New Medicine” construction index for medical schools has been built by employing meta-ethnography approach, which is not only based on massive literature and policy documents, but also based on the use of scientific theoretical construction methods to form the draft of the evaluation index system. Moreover, from the division of all literature subject units to the construction of three-level and two-level concepts, and then to the synthesis of first-level dimensions, the whole process of constructing the preliminary index system has been established through repeated analysis and thinking. It not only ensures the restoration of the original literature content, but also conforms to the theme of this study, seeks common ground while reserving differences, and forms a new synthesis result.

Secondly, after strict screening and verification on Delphi expert panel composition, the questionnaire including the assessment part of preliminary indicator system content, experts’ self-assessment of familiarity was distributed to the selected experts. Then two rounds of consulting process was conducted to meet the requirements involving Kendall’s coordination coefficient and index variation coefficient. In the end, a total of 11 primary indicators and 37 secondary indicators were obtained to proceed the following step.

Lastly, the establishment of weight coefficient of each indicator was obtained by employing AHP, and the weight value is determined by the importance value from experts based on Saaty’s scale. The result shows that CR < 0.1, so that it conforms with the expert consistency standard. According to the distribution of the final weight value, Curriculum System Reform of medical schools is imminent and has become the most important indicator, with the diversification of course formats, integration of industry and education, being the most important component. Besides, Medical Student Education and Training, Optimization and Adjustment of the Structure of Academic programs, Faculty Competence Development, Discipline System Construction also have strongly effect on the quality of “New Medicine” construction.

## 6 Limitations and suggestions for future research

Several limitations of this study should be acknowledged. Firstly, the selection of Delphi experts was mainly concentrated in specific regions and professional fields in China, which may lead to selection bias and affect the representativeness of the consensus results. Secondly, the indicator system constructed in this study is based on the Chinese educational context and cultural background, and its applicability in other regions or cross-cultural environments is limited. Thirdly, although the integrated method of meta-ethnography, Delphi and AHP was adopted, there was still some subjectivity in the integration of multiple methods, which may affect the interpretation of indicators and the distribution of weights.

Future research can expand the scope of Delphi experts to improve the robustness of the results, and conduct cross-regional comparisons to test the universality of the system. In addition, it is necessary to conduct pilot applications in actual medical colleges to verify the reliability, validity and operability of the indicator system. Further research should also explore how policymakers and education administrators can incorporate the system into quality assurance mechanisms, such as developing supporting implementation guidelines or digital tools to promote the implementation and promotion of the indicator system.

## Data Availability

The raw data supporting the conclusions of this article will be made available by the authors, without undue reservation.
