# Supplementary material for: Developing evaluation index system for “new medicine” construction in Chinese medical schools—meta-ethnography and Delphi-AHP approach
Source: Front Med (Lausanne). 2026 Jan 7;12:1717165. doi: 10.3389/fmed.2025.1717165 (PMC12819698; doi:10.3389/fmed.2025.1717165)
Supplement: Supplementary file 1 [file Table_1.docx]

Supplementary Material

# Supplementary Table

**TABLE 6** The weights coefficient of each indicator

| **Evaluation dimensions** | **First-level indicators** | **Weight coefficient** | **Second-level indicators** | **Weight coefficient** | **Combination coefficient** |
| --- | --- | --- | --- | --- | --- |
| Conceptual cognition | A Conceptual cognition of “New Medicine ” construction | 0.0460 | A1 Understanding the background of the “New Medicine” construction | 0.1724 | 0.0091 |
|  |  |  | A2 Understanding and grasping the essence of the “New Medicine” | 0.3338 | 0.0154 |
|  |  |  | A3 Understanding levels of innovation and adaptability based on the reality of ones’ own school | 0.4938 | 0.0227 |
| Medical talents cultivation | B Optimization and adjustment of the structure of  academic programs | 0.1530 | B1 The level of adjustment and quality improvement in the current programs | 0.1848 | 0.0283 |
|  |  |  | B2 The establishment and development of high-demand medical programs | 0.3006 | 0.0460 |
|  |  |  | B3 The establishment of new specialized emerging programs that align with the actual conditions of ones’ own school | 0.4396 | 0.0673 |
|  |  |  | B4 Establishment of an emerging medical professional certification system aligned with international standards | 0.075 | 0.0115 |
|  | C Disciplinary system construction | 0.1020 | C1 The construction of emerging interdisciplinary fields | 0.2993 | 0.0305 |
|  |  |  | C2 The integration of group medicine in the context of “holistic health”and “comprehensive wellness” | 0.2557 | 0.0261 |
|  |  |  | C3 The construction of interdisciplinary and transdisciplinary sustainable development mechanisms | 0.4450 | 0.0454 |
|  | D Curriculum System Reform | 0.1790 | D1 The establishment of various types of courses, including specialized courses, general education courses, and interdisciplinary courses | 0.2207 | 0.0395 |
|  |  |  | D2 The course content covers concepts such as health awareness, interdisciplinary integration, and the benevolence of a healer, as well as fostering students’ divergent and innovative thinking | 0.2787 | 0.0499 |
|  |  |  | D3 The diversification of course formats, integration of industry and education, and the use of innovative teaching methods such as virtual simulation experiments | 0.1755 | 0.0314 |
|  |  |  | D4 The establishment of a multi-dimensional evaluation model for courses | 0.1603 | 0.0287 |
|  |  |  | D5 The level of improvement in teaching quality after the curriculum reform | 0.1649 | 0.0295 |
|  | E Medical Student Education and Training | 0.1550 | E1 The improvement rate of medical humanities literacy among medical students | 0.0614 | 0.010 |
|  |  |  | E2 The professional competency development status of medical students | 0.2708 | 0.0420 |
|  |  |  | E3 The improvement rate of professional competence and innovation ability among medical students | 0.4169 | 0.0646 |
|  |  |  | E4 Hierarchical and classified training system for medical talents | 0.1093 | 0.0169 |
|  |  |  | E5 Diverse evaluation mechanisms for medical students | 0.1415 | 0.0219 |
| Faculty development | F Faculty  competence  development | 0.1340 | F1 The teaching skill level of teachers | 0.2377 | 0.0319 |
|  |  |  | F2 The construction of an innovative, interdisciplinary faculty team | 0.3392 | 0.0455 |
|  |  |  | F3 Status of establishing a teacher classification evaluation mechanism | 0.2288 | 0.0307 |
|  |  |  | F4 Level of optimization of the faculty structure | 0.1943 | 0.0260 |
| Organizational platform development | G The construction of organizational systems | 0.0500 | G1 The establishment of specialized interdisciplinary organizational structures and operational mechanisms | 0.4814 | 0.0241 |
|  |  |  | G2 The interactivity, permeability, and openness between the existing various organizational departments | 0.5186 | 0.0259 |
|  | H The construction and operation of the platform (physical platform and virtual platform) | 0.0530 | H1 The construction and operation of clinical skills training demonstration centers or simulation teaching centers | 0.2915 | 0.0154 |
|  |  |  | H2 The establishment and operation of innovation bases and experimental platforms for basic medical research | 0.2667 | 0.0141 |
|  |  |  | H3 The construction and operation of interdisciplinary teaching and experimental platforms | 0.2608 | 0.0138 |
|  |  |  | H4 The construction and operational status of the Telemedicine Center and the Virtual Simulation Experimental Teaching Center | 0.181 | 0.0096 |
| Social services | I Social health services | 0.0560 | I1 The number of trained medical personnel in various levels of healthcare institutions (especially in grassroots healthcare service institutions) | 0.3321 | 0.0186 |
|  |  |  | I2 The status of scientific and technological application in the integration of basic medical science and clinical medical science | 0.3336 | 0.0187 |
|  |  |  | I3 The ability to respond to public health challenges | 0.3343 | 0.0187 |
| Communication and Cooperation | J International communication and cooperation | 0.0540 | J1 The number and effectiveness of joint student training programs with international organizations | 0.2577 | 0.0139 |
|  |  |  | J2 Number of international medical aid and health cooperation services | 0.2538 | 0.0137052 |
|  |  |  | J3 The number of shared projects for medical education resources and technology | 0.4885 | 0.026379 |
| Feedback mechanism | K Stakeholder feedback mechanism | 0.0190 | K1 The establishment of effective feedback channels and systems for students, teachers, school administrators, employers, and recipients of social health services | 1 | 0.0190 |
